# An involvement of neurokinin-1 receptor in FcεRΙ-mediated RBL-2H3 mast cell activation

**DOI:** 10.1007/s00011-012-0523-x

**Published:** 2012-07-21

**Authors:** Xiaoyun Fang, Hua Hu, Jianhui Xie, Haiyan Zhu, Dongmei Zhang, Wei Mo, Ruxin Zhang, Min Yu

**Affiliations:** 1 The Key Laboratory of Molecular Medicine, Ministry of Education, Shanghai, 200032 People’s Republic of China; 2 Department of Biochemistry and Molecular Biology, Shanghai Medical College, Fudan University, Shanghai, 200032 People’s Republic of China; 3 Department of Otolaryngology, Huadong Hospital of Shanghai Medical College, Fudan University, Shanghai, 200032 People’s Republic of China; 4 Department of Otolaryngology, EENT Hospital of Shanghai Medical College, Fudan University, Shanghai, 200032 People’s Republic of China

**Keywords:** Neurokinin-1 receptor, Mast cell, FcεRΙ, Signaling pathway

## Abstract

**Objective and design:**

To determine whether the neurokinin-1 receptor (NK1R) plays a role in the activation of RBL-2H3 mast cells after FcεRΙ aggregation.

**Materials and methods:**

NK1R expression in RBL-2H3 cells was inhibited by small hairpin RNA (shRNA) against NK1R, and determined by western blotting. For activation, both NK1R knockdown and control RBL-2H3 cells were sensitized by dinitrophenol (DNP)-specific IgE and stimulated with the antigen DNP-bovine serum albumin (BSA). Following the activation of RBL-2H3 cells, monocyte chemoattractant protein (MCP-1) production and intracellular calcium flux were monitored by ELISA and confocal microscopy assay, respectively. For investigation of the signaling mechanism, phosphorylation of mitogen-activated protein kinases (MAPKs) after RBL-2H3 cell activation was assessed by western blotting.

**Results:**

shRNA-NK1R mediated an effective inhibition of NK1R expression in RBL-2H3 cells. Protein production of MCP-1 was reduced by more than 55 % in NK1R knockdown RBL-2H3 cells compared with control RBL-2H3 cells. In addition, both calcium mobilization and phosphorylation levels of MAPKs (Erk1/2, JNK, and p38) after DNP-BSA stimulation (via FcεRΙ) were decreased due to the inhibition of NK1R expression.

**Conclusion:**

NK1R is required for the activation of RBL-2H3 cells following FcεRΙ engagement and involved in the regulation of MAPK signaling pathways.

**Electronic supplementary material:**

The online version of this article (doi:10.1007/s00011-012-0523-x) contains supplementary material, which is available to authorized users.

## Introduction

Mast cells are major effectors in allergic diseases. The activation of mast cells leads to the release of various inflammatory mediators, including vasoactive agents, cytokines, and chemokines, contributing to the disease [1]. The key receptor distributed on the surface of mast cells and responsible for their activation is the IgE receptor, FcεRΙ [2]. In a typical allergic response, polyvalent antigens cross-link IgE-bearing FcεRΙ molecules on mast cells. This causes the activation of immunoreceptor tyrosine-based activation motifs (ITAMs) in tails of FcεRΙ and thus initiates intracellular signaling cascades that ultimately result in the activation of mast cells [1, 3]. Many signal molecules have been shown to be essential for FcεRΙ-mediated mast cell activation, such as Src family kinases Lyn and Fyn [4–7]. However, the precise mechanism underlying mast cell activation remains far from fully understood.

NK1R is a widely expressed G protein-coupled receptor (GPCR) and the preferred receptor for neuropeptide substance P (SP) [8]. It has been revealed to be involved in various biologic responses, including pain transmission, neuroimmune modulation, exocrine secretion, and inflammation [9–13]. The close relationship between NK1R and inflammation has evidenced by the marked elevation of NK1R expression in several kinds of inflammatory diseases, such as asthma [14], acute pancreatitis [15–18], and abdominal cell adhesion formation [19, 20]. Functional expression of NK1R on bone marrow-derived mast cells (BMMCs) induced by IL4 and SCF further suggests the implications of NK1R in inflammation [21]. However, the detailed role of NK1R in allergic disorders remains unclear. Given that previous reports have demonstrated that NK1R is expressed in the RBL-2H3 cell line, a rat mast cell line with the properties of mucosal mast cells [22, 23], determining whether NK1R plays a role in FcεRΙ-induced activation of RBL-2H3 cells, will be of significance.

In this work, by inhibiting the expression of NK1R in RBL-2H3 cells, we explored potential functions that NK1R exerts in FcεRΙ-induced RBL-2H3 cell activation in an attempt to provide new insights into the mechanism underlying allergic diseases.

## Materials and methods

### Cell culture conditions

RBL-2H3 cell line [24] was maintained as monolayer cultures in Earle’s modified Eagle’s medium (MEM), supplemented with 10 % heat-inactivated fetal bovine serum (FBS; HyClone), 2 mM glutamine, 100 U/ml penicillin, and 100 μg/ml streptomycin at 37 °C in a humidified incubator under 5 % CO_2_. All cell culture reagents were obtained from Gibco except where otherwise indicated.

### Plasmid construction and transfection

Two different small hairpin RNAs (shRNAs) against NK1R were designed. NK1R-shRNA (NK1R-shRNA1 and NK1R-shRNA2) constructs were produced as described previously [25]. The sequences of the oligonucleotides homologous to a 21-nucleotide segment of NK1R were: (1) 5′-GCCAGUAUCUACUCCAUGAUU-3′ for NK1R-shRNA1 and 5′-CCUACAUCAACCCAGAUCUUU-3′ for NK1R-shRNA2. A scramble shRNA (negative control, Con-shRNA) was also designed. For transfection, plasmids (2.5 μg) were transfected into RBL-2H3 cells using SuperFect reagent (Qiagen) according to the manufacturer’s protocol. The transfected cells were harvested for further study 36 h later.

### Western blotting

For determining the expression of NK1R, RBL-2H3 cells (10^6^) were lysed with 100 μl of lysis buffer (2 % SDS, 50 mM Tris–HCl pH 6.8, 4 μg/ml aprotinin, 20 μg/ml leupeptin, 2 μg/ml pepstatin, 2 μg/ml antipain, and 200 μg/ml phenylmethylsulfonyl fluoride). The lysates were boiled for 10 min and protein concentrations were determined using a bicinchoninic acid (BCA) protein assay kit (Pierce Biotechnology) according to the manufacturer’s instructions. The cell lysates were then mixed with 5× sample buffer and fractionated onto 10 or 12 % SDS-polyacrylamide gel electrophoresis (PAGE) for NK1R analysis. After SDS-PAGE gels were run, proteins were blotted onto a polyvinylidene difluoride (PVDF) membrane (Roche Applied Science) for 90 min at 350 mA. The membrane was then blocked in TBS-Tween (0.1 %) buffer containing 5 % skim milk or bovine serum albumin (BSA, Amresco) for 2 h at room temperature (RT) followed by probing with anti-NK1R antibody (1:500–1:1,000, overnight, 4 °C; Pierce Biotechnology). The membrane was washed and incubated with goat anti-rabbit IgG conjugated to horseradish peroxidase (1:2,000, 1 h, RT; Santa Cruz Biotechnology). Immunoreactive bands were visualized using enhanced chemiluminescence (ECL). The membrane was reprobed with anti-GAPDH antibody after incubation with stripping buffer (100 mM 2-mercaptoethanol, 2 % SDS, 62.5 mM Tris–HCl pH 6.8) for 30 min at 50 °C to control for loading. Densitometric analysis was performed by ImageJ software. Densitometry was measured in arbitrary densitometry units (ADU).

### Measurement of calcium flux

RBL-2H3 cells (2 × 10^5^) were sensitized with 0.1 μg/ml anti-dinitrophenol (DNP)-IgE (SPE-7 clone; Sigma-Aldrich) overnight and washed twice with Tyrode’s buffer (135 mM NaCl, 5 mM KCl, 1.8 mM CaCl_2_, 1.0 mM MgCl_2_, 5.6 mM glucose, 20 mM HEPES, and 1 mg/ml BSA at pH 7.4). The cells were then loaded with 4 μM Fluo3-AM (Dojindo, Japan) in Tyrode’s buffer for 45 min at 37 °C in a 5 % CO_2_ incubator. To monitor the calcium flux, Fluo3-loaded cells were stimulated with the antigen DNP-BSA (10 ng/ml) for the indicated lengths of time, and single-wavelength measurements of Ca^2+^-bound Fluo3 at 520 nm (488 nm excitation) were performed on a laser scanning confocal microscope (Leica Microsystems, Heidelberg, Germany). Images were recorded and analyzed using the “LAS AF Lite” software package (Leica), and the elevation in fluorescence intensity of Ca^2+^-bound Fluo3, proportional to the [Ca^2+^], was calculated after subtracting the background fluorescence.

### ELISA for MCP-1 production

RBL-2H3 cells (3 × 10^6^) incubated overnight in complete MEM containing anti-DNP IgE (0.1 μg/ml) were washed and stimulated with DNP-BSA (10 ng/ml) for 4 h. Cell-free supernatants were collected and secreted MCP-1 was determined using a rat-specific MCP-1 ELISA detection kit (Invitrogen).

### MAPK assays

DNP-BSA-induced phosphorylation of mitogen-activated protein kinases (MAPKs) was determined by western blotting. RBL-2H3 cells were sensitized and starved overnight in serum-free MEM containing anti-DNP-IgE (0.1 μg/ml) and BSA (2 %). Stimulation was initiated by the addition of DNP-BSA (10 ng/ml) in Tyrode’s buffer at 37 °C. The reaction was stopped by placing the cells on ice. To determine the activities of MAPK molecules, the whole cell lysates were prepared and subjected to western blotting as described above. Additional protease inhibitors including 60 mM β-glycerophosphate, 2 mM sodium orthovanadate, and 10 mM sodium fluoride were added in the lysates. Anti-phospho-Erk1/2, JNK, and p38 antibodies (phospho-MAPKs) were used. To control for loading, membranes were stripped and reprobed with antibodies to total Erk1/2, JNK, and p38 (MAPKs). The intensities of the phospho-MAPKs were normalized to total MAPKs to correct for any differences in loading. All antibodies were purchased from Cell Signaling Technology unless otherwise noted and used at recommended concentrations. Aprotinin, leupeptin, pepstatin, antipain, phenylmethylsulfonyl fluoride, sodium orthovanadate, and sodium fluoride were from Sigma-Aldrich.

### Statistical analysis

All data were analyzed with Prism 5 software (GraphPad Software). Data were considered statistically significant when a *P* value was less than 0.05, obtained with a two-tailed *t* test.

## Results

### shRNA-mediated knockdown of NK1R expression in RBL-2H3 cells

**Fig. 1:**
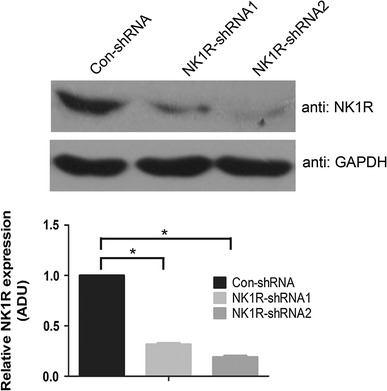
An effective inhibition of NK1R expression mediated by the shRNA in RBL-2H3 cells. *Upper panel* wild-type RBL-2H3 cells were transfected with Con-shRNA, NK1R-shRNA1, and NK1R-shRNA2 constructs. The transfected cells were harvested 36 h later and the effect of these three shRNA constructs on the expression of NK1R was analyzed by western blotting with anti-NK1R antibody. Representative blots from three independent experiments are shown. *Lower panel* densitometry analysis of NK1R expression is shown. Values normalized to GAPDH expression are represented as mean ± SD (*n* = 3) and are expressed in arbitrary densitometry units (ADU). *P < 0.05 as compared with control RBL-2H3 cells, Student’s *t* test

To determine whether NK1R plays a role in FcεRΙ-mediated RBL-2H3 cell activation, we constructed two shRNAs against NK1R (NK1R-shRNA1 and NK1R-shRNA2) and transfected them into wide-type RBL-2H3 cells to inhibit the expression of NK1R. Negative control plasmids containing scrambled shRNA (Con-shRNA) were also transfected. NK1R protein expression in RBL-2H3 cells after 36 h of transfection was assessed by western blotting (Fig. 1). Expression of NK1R-shRNA1 and NK1R-shRNA2 led to reductions in NK1R expression of 68.43 ± 2.41 and 81.1 ± 2.49 %, respectively, in RBL-2H3 cells compared with Con-shRNA. This indicates an effective knockdown of NK1R expression in RBL-2H3 cells by either NK1R-shRNA1 or NK1R-shRNA2. Hence, we named RBL-2H3 cells expressing NK1R-shRNA1 or NK1R-shRNA2 for NK1R knockdown RBL-2H3 cells and expressing Con-shRNA for control RBL-2H3 cells.

### NK1R is required for the FcεRΙ-evoked activation of RBL-2H3 cells

**Fig. 2:**
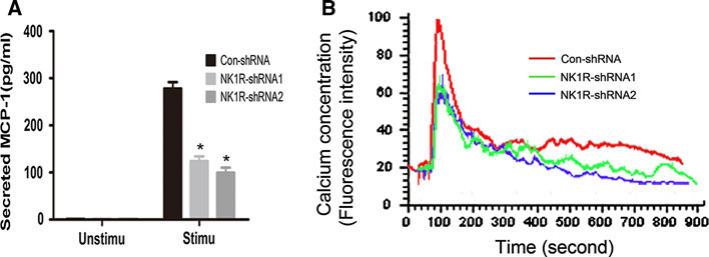
NK1R promotes FcεRΙ-induced expression of MCP-1 and calcium mobilization in RBL-2H3 cells. **a** Indicated RBL-2H3 cells were sensitized overnight with anti-DNP IgE (0.1 μg/ml) and stimulated with (*Stimu*) or without (*Unstimu*) DNP-BSA (10 ng/ml) for 4 h at 37 °C. Amounts of released MCP-1 in cell-free supernatants were determined by ELISA. Results are representative of three independent experiments. *P < 0.05 as compared with control RBL-2H3 cells, Student’s *t* test. **b** Sensitized RBL-2H3 cells were loaded with 4 μM Fluo3-AM and changes in dye fluorescence with time were monitored by confocal microscopy after the addition of DNP-BSA (10 ng/ml). Calcium flux is indicated by the antigen–response curve over time. Values are presented as the mean fluorescence intensity of Ca^2+^-bound Fluo3 of at least 20 cells. Results of two independent experiments are shown in each panel

Cytokine gene expression and calcium mobilization are two remarkable events in mast cells after FcεRΙ aggregation. To evaluate the effect of NK1R on cytokine gene expression in RBL-2H3 cells following FcεRΙ stimulation, amounts of released MCP-1 were determined by ELISA. As shown in Fig. 2a, a significant reduction of MCP-1 expression was observed in NK1R knockdown RBL-2H3 cells (124.1 ± 17.7 for NK1R-shRNA1, and 99.5 ± 18.6 for NK1R-shRNA2) relative to control RBL-2H3 cells (278.2 ± 23.5 for Con-shRNA). We also monitored calcium flux in both control and NK1R knockdown RBL-2H3 cells following FcεRΙ aggregation (Fig. 2b). RBL-2H3 cells expressing either NK1R-shRNA1 or NK1R-shRNA2 showed decreased calcium mobilization compared with RBL-2H3 cells expressing Con-shRNA. These results strongly indicate an essential role of NK1R in FcεRΙ-evoked RBL-2H3 cell activation.

### NK1R knockdown results in defective activation of MAPKs

**Fig. 3:**
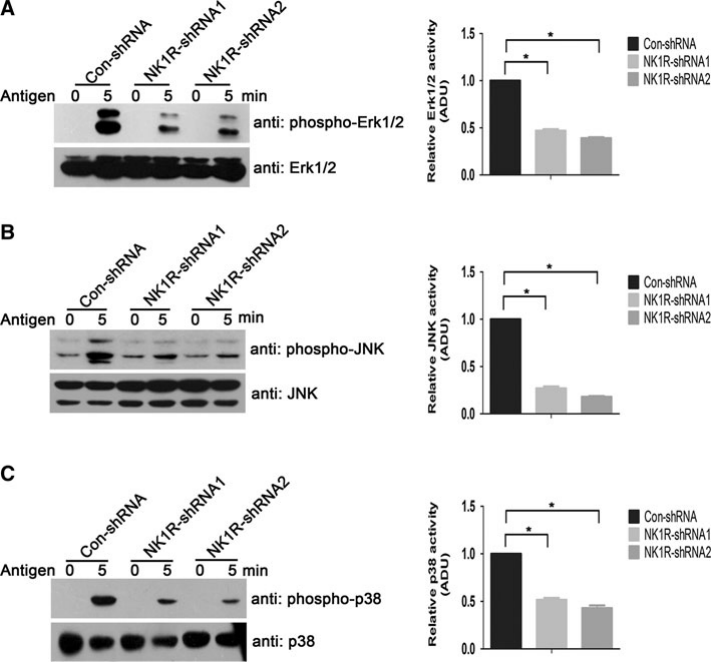
NK1R contributes to the phosphorylation of MAPKs following FcεRI aggregation in RBL-2H3 cells. Sensitized RBL-2H3 cells were starved and stimulated with or not with DNP-BSA (10 ng/ml) for 5 min. The whole cell lysates were immunoblotted with phospho-specific antibodies for Erk1/2 (**a**), JNK (**b**), and p38 (**c**), respectively. To control for loading, the blots were stripped and reprobed with anti-total Erk1/2, JNK, and p38 antibodies, respectively. Representative blots of at least three independent experiments are shown. The levels of phospho-MAPKs (at 5 min) were normalized to the expression of MAPKs and qualified by densitometry in ADU. The phosphorylation levels of Erk1/2 decreased by 52.99 ± 2.84 % for NK1R-shRNA1 and 60.96 ± 1.88 % for NK1R-shRNA2; of JNK decreased by 73.08 ± 3.66 % for NK1R-shRNA1 and 81.97 ± 1.33 % for NK1R-shRNA2; and of p38 decreased by 48.33 ± 3.51 % for NK1R-shRNA1 and 57 ± 4.58 % for NK1R-shRNA2, as compared with Con-shRNA. Values are expressed as mean ± SD (*n* = 3). *P < 0.05 as compared with the control RBL-2H3 cells, Student’s *t* test

Efforts were made to explore the effect of NK1R on MAPK signaling downstream of the FcεRΙ in RBL-2H3 cells. After FcεRΙ stimulation, phosphorylation levels of MAPKs were determined by western blotting. For phospho-Erk1/2, phosphorylation signals were hardly detected under basal conditions in both control and NK1R knockdown RBL-2H3 cells. After the antigen DNP-BSA treatment for 5 min, control RBL-2H3 cells showed a marked increase in levels of phospho-Erk1/2. However, a much lower elevation of phosphorylation levels of Erk1/2 were observed in NK1R knockdown RBL-2H3 cells compared with control RBL-2H3 cells (Fig. 3a). Similar tendencies were observed on both phospho-JNK (Fig. 3b) and phospho-p38 (Fig. 3c). This significantly inhibited phosphorylation of MAPKs, which is attributable to the down-regulation of NK1R expression, indicates an involvement of NK1R in the regulation of MAPK signaling after FcεRΙ aggregation in RBL-2H3 cells.

## Discussion

In the present study, we demonstrate that NK1R promotes FcεRΙ-evoked MCP-1 mRNA expression and calcium mobilization in RBL-2H3 cells. Furthermore, our results suggest that NK1R is required for the initiation of rapid, full activation of MAPKs upon FcεRΙ aggregation in RBL-2H3 cells. Previous studies have revealed an involvement of NK1R in various inflammatory disorders, but little is known about the role of NK1R in FcεRΙ-mediated allergic reactions. Our data provide the first evidence for functional roles of NK1R in the activation of RBL-2H3 cells following FcεRΙ aggregation.

MCP-1 is one of chemokines produced by mast cells after FcεRΙ activation [26, 27]. Given that the expression of MCP-1 was significantly reduced in Fyn-deficient BMMCs [28], Fyn kinase has been reported to be essential for the production of MCP-1 after FcεRΙ aggregation in mast cells. Our finding that NK1R knockdown RBL-2H3 cells showed decreased MCP-1expression after antigen stimulation (Fig. 2a) suggests that NK1R is also required for the production of MCP-1 and that there perhaps exist cross-talk between NK1R and Fyn in FcεRΙ signaling. Moreover, the decreased phosphorylation levels of JNK (Fig. 3b) and p38MAPK (Fig. 3c) observed in NK1R knockdown RBL-2H3 cells were also similar to that reported in Fyn-deficient BMMCs [28]. Activities of MAPKs are required for the MCP-1 gene expression in mast cells [29]. Based on these results, it is possible that NK1R promotes the FcεRΙ-induced MCP-1 expression through Fyn kinase. Actually, in mouse pancreatic acinar cells, NK1R has been demonstrated to mediate the production of MCP-1 via Src family kinases following the stimulation of SP [30]. More experiments are needed to be carried out to delineate the relationship between NK1R and Fyn in mast cells.

As with the reduced MCP-1 expression, we found that calcium flux was also inhibited as a consequence of the knockdown of NK1R expression in RBL-2H3 cells (Fig. 2b). This is as we expected, because the release of inflammatory mediators is always coupled with the elevation of intracellular calcium concentration following the activation of mast cells [7, 26]. FcεRΙ-evoked calcium mobilization in mast cells involves the activation of phospholipase C (PLC) [26, 31]. The activated PLC is capable of inducing the production of diacylglycerol (DAG) and inositol 1,4,5-trisphosphate (IP3), both of which are required for calcium mobilization, leading to increased calcium concentration [32]. Previous studies have demonstrated that NK1R, as a GPCR, is coupled to Gq/11 receptor. This receptor is associated with PLC [33]. These clues allow us to assume that the inhibition of NK1R expression in RBL-2H3 cells may impair the interaction of Gq/11 with PLC, resulting in reduced activity of PLC followed by decreased calcium flux after FcεRΙ stimulation. This assumption might be reasonable considering that the NK1R expressed in RBL-2H3 cells is in its full-length form (407aa). In fact, NK1R has two naturally occurring forms: a full-length NK1R (407aa) isoform and a truncated NK1R (311aa) isoform lacking 96-aa residues in the carboxyl terminus [34]. The absent C-terminal domain is required for the coupling to Gq/11 receptor, making the truncated NK1R disassociated with PLC [8, 33] and unable to prime calcium mobilization by itself.

The functional role of NK1R in FcεRΙ signaling is further confirmed by the observation that the phosphorylation of Erk1/2 was defective in NK1R knockdown RBL-2H3 cells (Fig. 3a). This, in addition to similar defects found on phospho-JNK (Fig. 3b) and phospho-p38MAPK (Fig. 3c), indicates a critical role of NK1R in MAPK signaling downstream of the FcεRΙ in RBL-2H3 cells. The activation of NK1R by DNP-BSA (via FcεRΙ) is virtually identical to that by SP, resulting in the expression of MCP-1 as well as the phosphorylation of MAPKs [35]. Previous investigations of the signaling mechanism have revealed that SP activation of NK1R induces the phosphorylation of Erk1/2 involves β-arrestin/src or β-arrestin2 [36]. Whether this mechanism is reasonable to explain the role of NK1R in FcεRΙ signaling awaits further exploration. In addition, the question of how NK1R is activated and then involved in the following signaling events after FcεRΙ cross-linking is still elusive and needs more attention in further study.

In conclusion, our data suggest that NK1R functions as a regulator in the activation of RBL-2H3 cells through FcεRΙ signaling. In this way, they contribute to the understanding of the mechanisms underlying allergic diseases.

## Electronic supplementary material

Below is the link to the electronic supplementary material.

Supplementary material 1 (DOC 319 kb)
