# Supplementary material for: An involvement of neurokinin-1 receptor in FcεRΙ-mediated RBL-2H3 mast cell activation
Source: Inflamm Res. 2012 Jul 21;61(11):1257–63. doi: 10.1007/s00011-012-0523-x (PMC3472057; doi:10.1007/s00011-012-0523-x)
Supplement: Supplementary file 1 — Supplementary material 1 (DOC 319 kb) [file 11_2012_523_MOESM1_ESM.doc]

Dear Prof. Andras Falus,

Many thanks for your comments on our manuscript entitled ''An involvement of neurokinin-1 receptor in FcεRΙ-mediated RBL-2H3 mast cell activation'' (IR-2012-0104). We appreciate the valuable comments on our work. We have revised our manuscript according to reviewers' comments and some changes in our manuscript are highlighted in red color. Our specific responses (point to point) to the reviewer’s comments are listed as follows.

Reviewer 1:

*General comments:
1. Though the manuscript looked at several pieces of evidences, including MCP-1 expression, calcium influx and MARK phosphorylation, it is unknown whether those events are really connected;*

**Response:** **1)** Yes, although MCP-1 expression, calcium flux and MAPK phosphorylation are all remarkable events after FcεRΙ aggregation in mast cells, whether they have direct connection in this process remains elusive. Nevertheless, whether these events have direct connections or not does not affect our conclusion that NK1R is involved in FcεRΙ-mediated mast cell activation, since NK1R knockdown RBL-2H3 mast cells showed significantly defective MCP-1 expression, calcium flux and MAPK phosphorylation after FcεRΙ aggregation. **2)** On the other hand, these events are really closely related to each other. As documented repeatedly, PLCγ is activated in many signaling pathways, such as epidermal growth factor receptor signaling and FcεRΙ signaling. Activated PLCγ is capable of cleaving phosphatidylinositol-4,5-bisphosphate (PIP2) into inositol 1,4,5-trisphosphate (IP3) and diacylglycerol (DAG), leading to the elevation of intracellular calcium concentration . Moreover, as a second messenger, DAG is needed for the activation of protein kinase C (PKC), which has been reported to be able to activate MAP kinases (MAPK) via Ras in T- and B lymphocytes . MAPK signaling pathway controls the production of a majority of cytokines and chemokines, including MCP-1. The above signaling cascade has been partly summarized by Nishizuka,Y. and we cited here shown as follows (Image 1):

**Image 1**

*2. It will be much more informative if MCP-1 was quantified by ELISA instead of western blotting or RT-PCR;*

**Response:** Thanks you for the comment. Yes, we have qualified MCP-1 expression by ELISA (Fig. 2a). The result got by ELISA was similar to that got by RT-PCR, indicating that NK1R influenced MCP-1 expression in response to FcεRΙ activation not only at RNA level, but also at protein level. Therefore, we replaced our RT-PCR result with ELISA result in Fig. 2a.

**Fig. 2a**: Indicated RBL-2H3 cells were sensitized overnight with anti-DNP IgE (0.1μg/ml) and stimulated with (stimu) or without (unstimu) DNP-BSA (10 ng/ml) for 4 h at 37°C. Amounts of released MCP-1 in cell-free supernatants were determined by ELISA. Results are representative of three independent experiments. *, *P*<0.05 as compared with control RBL-2H3 cells, Student’s *t* test.

*3. No information on the reproducibility of this experiment was provided;*

**Response:** We have indicated the reproducibility of every experiment in “Figure Captions” section, where we marked this information in red color.

*Specific comments:
1. The abstract needs to be written since current one did not provided sufficient data in the result section,*

**Response:** Thank you for the comment. We have re-written the abstract making it covering all necessary information mentioned in the result section.

*2. The keywords need to be re-selected, the name of cell line should not be used as key word.*

**Response:** Thank you for the comment. We have re-selected the key words.

*3. The discussion needs to be tune down.*

**Response:** Thank you for the comment. We have reduced the passage of discussion section.

*4. Figures are too small to be viewed*

**Response:** Thank you for the comment. We have adjusted figures' sizes to the requirement of Inflammation Research .

Reviewer 2:

*Major comments:
1. The loading of the cells and the detection of the calcium signals were carried out in Tyrode's buffer. What is the source of the ligand for NK1R under these stimulation conditions? How can the receptor be activated?*

**Response:** **1)** Actually, we did not stimulate NK1R with its specific ligand in this study. Our aim is to investigate whether NK1R is involved in FcεRΙ signaling, we thus activated IgE-bearing FcεRΙ by the antigen DNP-BSA to initiate FcεRΙ signaling in mast cells for an attempt to establish allergy model. Based on this model, we explored the role of NK1R in FcεRΙ signaling by inhibiting the expression of NK1R. Hence, we paid no attention on the ligand for NK1R in current study. **2)** Based on our experiment data, it’s still beyond our knowledge to understand how NK1R is activated when FcεRΙ is cross-linked by DNP-BSA. Although NK1R can be activated by its preferred ligand substance p (SP) and their interaction has been reported to be able to induce various clinical diseases including inflammation , our experiments did not use SP to stimulate cells (as explained above). One possibility that results in NK1R activation may exist in the mobility of lipid raft. Field,K.A. has demonstrated that the plasma membrane could form specialized regions (membrane domains or lipid raft) following FcεRΙ aggregation ; while NK1R has been suggested to localize to the plasma membrane microdomains . NK1R maybe link with FcεRΙ when membrane domains flux in response to the antigen stimulation followed by the activation of NK1R. More data should be complemented to understand the precise mechanism of NK1R activation in FcεRΙ signaling and it is really an attractive area for us to further explore.

*2. The authors should test which is the ligand for the receptor in their studies. What happens if they use serum-free medium?*

**Response:** About the question of the ligand for NK1R, we have explained above. What happens if use serum-free medium? In fact, before we activated cells, cells would be starved with serum-free medium overnight and then stimulated with the antigen DNP-BSA diluted with Tyrode's buffer (serum-free). The whole process is not involved with serum.

*3. One of the major functions of mast cells is the release of histamine. How does NK1R modulate this process in the presence of IgE/antigen?*

**Response:** Thanks for your comment. Yes, we have performed β-hexominidase assay to assess the role of NK1R in the process of FcεRΙ-evoked mast cell degranulation and the results are showed as follows (Image 2). Inconsistent with the decreased calcium flux, degranulation was largely unaffected in NK1R knockdown RBL-2H3 cells compared with control RBL-2H3 cells. On one hand, this suggests that degranulation and calcium mobilization may be controlled by separate signaling pathways in activated mast cells and that NK1R acts selectively. Actually, Valentino Parravicini et al. have reported that Fyn-deficiency BMMC showed normal calcium flux, but significantly inhibited degranulation following FcεRΙ stimulation, indicating independent signaling pathways in charge of calcium mobilization and degranulation. Our results thus further confirm the opinion of Valentino Parravicini et al. On the other hand, the cell line we used may result in such inconsistency. RBL-2H3 cell line may not be sensitive enough to the inhibition of NK1R expression, causing difficult to detect the change of degranulation. The significant difference in degranulation between NK1R knockdown and control mast cells may be observed if use primary cells, such as BMMC. Anyway, extra experimental data are required before we can clearly explain why calcium flux and degranulation show different tendency in NK1R knockdown RBL-2H3 mast cells following FcεRΙ activation.

**Image 2:** (A) Sensitized RBL-2H3 cells were cultured with either DNP-BSA or (B) PMA plus ionomycin (set for positive control) for 1 h at 37°C. The release of β-hexosaminidase was determined as described previously . Results are representative of at least three independent experiments. Data are expressed as the mean ± SD. *P*>0.05 compared with control RBL-2H3 cells, Student’s *t* test.

*4. The authors should test whether NK1R has the same effect in other model systems (such as BMMC, other mast cell lines etc).*

**Response:** We agree with your comment. It is really a kind suggestion to test whether NK1R has the same effect in other mast cells, so that the results will be more convincing and may be more pronounced. However, it is not so convenient for us to carry out this experiment currently, since we have not ready-to-use BMMC or other mast cell lines at present. Anyway, our laboratory is improving the research platform to further investigate the detail mechanism underlying allergic reaction. Now that NK1R has been proved to be involved in the allergic process, we will keep persistent attention on its role in other mast cell lines and primary mast cells.

*5. The figures are not suitable for publication, Fig2 and Fig3 are hardly visible.*

**Response:** Thank you for the comment. Yes, we have adjusted the size of figures to meeting the requirement of Inflammation Research.

*6. Although the authors suggest a mechanism (via Fyn), however, they have not addressed it experimentally.*

**Response:** Given that our focus in this study is on the question whether NK1R is involved in FcεRΙ-evoked mast cell activation, the present work just made some preliminary exploration on the role of NK1R in FcεRΙ signaling and we did not carry out relative experiments to further investigate the relationship between NK1R and Fyn. Actually, it is the first evidence that NK1R plays a role in the activation of RBL-2H3 mast cells induced by FcεRΙ aggregation. More efforts are needed to figure out the precise signaling regulatory mechanism of NK1R in this process. In fact, although we have focused on substance p (SP, the preferred ligand for NK1R) , our laboratory paid attention on the role of NK1R in allergic reaction not long ago. Many details are still to be clarified and we will further explore the mechanism of NK1R functions in allergic reaction with more efforts. Anyway, based on our present data combined with previous reports, Fyn kinase may be really an attractive substrate for NK1R in FcεRΙ signaling as we have detailed in the discussion section.

*Minor comments:
1. Materials and methods, Page 8: What dilution was used for the phospho-MAPK antibodies?*

**Response:** We used TBS-Tween(0.1%) containing 5% BSA (w/v) to dilute phospho-MAPK antibodies.

*2. Discussion, Page 13: which isoform was targeted by the shRNAs?*

**Response:** We targeted the full-length isoform of NK1R in this study. Actually, it is supposed that rats express the full-length isoform of NK1R (407aa) solely. Therefore, although our antibody recognizes both truncated NK1R (311aa) and full-length NK1R (407aa), it can be sure that RBL-2H3 cells (from the rat) express 407aa NK1R. We have supplemented this information in the discussion section highlighted in red color.

**References**

1. Gu HH, Saito K, Klaman LD, Shen JQ, Fleming T, Wang YP, et al. Essential role for Gab2 in the allergic response. Nature. 2001; 412:186-90.

2. Nishizuka Y. Protein kinase C and lipid signaling for sustained cellular responses. FASEB journal : official publication of the Federation of American Societies for Experimental Biology. 1995; 9:484-96.

3. Hirasawa N, Santini F, Beaven MA. Activation of the mitogen-activated protein kinase/cytosolic phospholipase A2 pathway in a rat mast cell line. Indications of different pathways for release of arachidonic acid and secretory granules. J Immunol. 1995; 154:5391-402.

4. Wong CK, Tsang CM, Ip WK, Lam CW. Molecular mechanisms for the release of chemokines from human leukemic mast cell line (HMC)-1 cells activated by SCF and TNF-alpha: roles of ERK, p38 MAPK, and NF-kappaB. Allergy. 2006; 61:289-97.

5. Douglas SD, Leeman SE. Neurokinin-1 receptor: functional significance in the immune system in reference to selected infections and inflammation. Ann N Y Acad Sci. 2011; 1217:83-95.

6. Field KA, Holowka D, Baird B. Compartmentalized activation of the high affinity immunoglobulin E receptor within membrane domains. J Biol Chem. 1997; 272:4276-80.

7. Monastyrskaya K, Hostettler A, Buergi S, Draeger A. The NK1 receptor localizes to the plasma membrane microdomains, and its activation is dependent on lipid raft integrity. J Biol Chem. 2005; 280:7135-46.

8. Hu H, Zhang R, Fang X, Yu M, Yu S, Zhang J, et al. Effects of endogenous substance P expression on degranulation in RBL-2H3 cells. Inflammation research. 2011; 60:541-6.

Finally, many thanks again for your time and effort on our manuscript. We hope that the manuscript is acceptable for publication in Inflammation Research. If any further information is required, please let me know.

Thank you very much for your attention and consideration.

Best wishes,

Sincerely yours,

Min Yu Ph.D.

Professor of Biochemistry and Molecular Biology
Fudan University Shanghai Medical College
Shanghai 200032, People's Republic of China
E-mail: minyu@shmu.edu.cn
